# A Study of the Use of Iconic and Metaphoric Gestures with Motion-Based, Static Space-Based, Static Object-Based, and Static Event-Based Statements

**DOI:** 10.3390/bs12070239

**Published:** 2022-07-18

**Authors:** Omid Khatin-Zadeh, Danyal Farsani, Hassan Banaruee

**Affiliations:** 1 School of Foreign Languages, University of Electronic Science and Technology of China, Chengdu 610054, China; khatinzadeh.omid@yahoo.com; 2 Department of Teacher Education, Norwegian University of Science and Technology, 7491 Trondheim, Norway; 3 Department of English, American, and Celtic Studies, University of Bonn, 53115 Bonn, Germany; hassan.banaruee@uni-bonn.de

**Keywords:** iconic gesture, metaphoric gesture, motion-based, static space-based, static object-based, static event-based

## Abstract

In this article, we extend our previously suggested categorization of metaphors to literal statements, and categorize metaphorical and literal statements into four pairs of corresponding metaphorical and literal statements: (1) motion-based metaphorical/literal statements; (2) static space-based metaphorical/literal statements; (3) static object-based metaphorical/literal statements; (4) static event-based metaphorical/literal statements. Then, we report a study that investigated the use of metaphoric and iconic gestures with these corresponding categories during the retelling of a set of stories by a group of thirty participants. The participants listened to five audio short stories. Each story contained one statement of each metaphoric category and one statement of each literal category. After listening to each story, they retold it in their own language in front of a camera. The results showed that event-based metaphors and event-based literal statements were accompanied by the smallest number of metaphoric and iconic gestures. Furthermore, there was a significant similarity between each metaphorical category and its corresponding literal category in the number of gestures that were used with these categories. This similarity supports the idea that the mechanisms underlying the embodiment of metaphorical and literal statements are essentially similar.

## 1. Introduction

The differences between the mechanisms of understanding literal and metaphorical statements have been the subject of a large body of work in cognitive linguistics, cognitive psychology, and related fields. Metaphorical statements differ from literal ones because they do not directly refer to their intended meanings. To understand a metaphorical statement, the individual must look beyond the surface or literal meanings of words and figuratively interpret the statement [1,2]. In the literal statement *my hands are tied by the rope*, there is a direct relationship between the meanings of words and the literal meaning of the statement. On the other hand, in the metaphorical statement *my hands are tied by the new law*, there is no direct relationship between the meanings of words and the intended figurative meaning of the statement, as an actual concrete object does not tie the hands. This critical difference between metaphorical and literal statements has been the main reason behind a widely debated question: is there any difference between the cognitive processes involved in comprehending literal and metaphorical statements? To answer this question, several theories have been suggested by researchers, such as the pragmatic theory [3,4], the salience imbalance model [5], the class-inclusion theory [6,7], the structure-mapping theory [8], and the conceptual metaphor theory [9]. While some views hold that literal and metaphorical statements are comprehended through different mechanisms [8,10], others assume that mechanisms of comprehending literal and metaphorical statements are essentially similar when they are viewed from a class-inclusion perspective [7] or an embodied perspective [11]. The class-inclusion model of metaphor comprehension holds that literal and metaphorical class-inclusion statements are comprehended in the same way [6]. Embodiment theories hold that literal and metaphorical statements are embodied in a similar manner [11]. For example, the same sensorimotor networks involved in the processing of the literal phrase *grasping an object* are also involved in the metaphorical phrase *grasping an idea*.

In this study, we aimed to examine one specific possible similarity or difference between literal and metaphorical statements. We wanted to know if there was any difference or similarity between literal and metaphorical statements in the ways that gestures are used with these statements. To achieve this objective, we build on our previously suggested classification of metaphors [12]. We extend this classification to literal statements and make a comparison between each category of metaphors and its corresponding literal category. In addition, we specifically intended to examine the possible similarity or difference between each category of metaphors and its corresponding category of literal statements in the ways that gestures are used with these statements. Before describing the study’s methodology, we briefly overview our classification of metaphors [12] and then present a corresponding classification of literal statements. This section will be followed by a brief look at the iconic and metaphoric gestures that are used with literal and metaphorical statements.

## 2. A Classification of Metaphoric and Literal Statements

According to our classification [12], metaphors are grouped into four categories: (1) motion-based metaphors; (2) static space-based metaphors; (3) static object-based metaphors; (4) static event-based metaphors. In a motion-based metaphor, a static concept, which may be abstract, is described as a motion event [13]. The metaphor *time is moving fast* is an example of a motion-based metaphor. A static space-based metaphor describes a concept in terms of a location in the space. For example, the metaphor *people at the lowest level of society have serious problems* is a static space-based metaphor. In a static object-based metaphor, a given concept, which may be abstract, is described as a static concrete object. For example, the metaphor *there is a mountain of problems in this organization* describes the problems of an organization as a mountain. A static event-based metaphor describes a concept, which may be abstract, in terms of a static event. For example, the metaphor *this new technology is a revolution in our organization* is an example of a static event-based metaphor in which new technology is described as a static event (revolution).

Here, we present a classification of literal statements parallel to this classification of metaphors. Then, we report a study based on these parallel classifications of metaphorical and literal statements. As with the classification of metaphors, we classify literal statements into four categories: (1) motion-based literal statements; (2) static space-based literal statements; (3) static object-based literal statements; (4) static event-based literal statements. A motion-based literal statement describes the real movement of an object. The literal statement *the car is moving fast* is an example of such a statement. A static space-based literal statement describes the physical location of a concrete object in the environment. For example, the sentence *the car is under the bridge* describes the car’s location relative to other concrete objects in the physical environment. A static object-based literal statement represents a static concrete object in the real environment. The statement *the ten-story building is really big* is an example of such a statement. This sentence describes a concrete feature (big) of a physical object (building) in the real environment. A static event-based literal statement describes a concretely perceivable non-motion event in the real environment. This event may take place in a short or over a long period of time. The statement *the paper is burning* is an example of such a sentence. Like static event-based metaphors, static event-based literal statements may involve some degree of motion, but these motions are not central to the meaning of the sentence. Table 1 presents a clear picture of these parallel categories of metaphorical and literal statements.

The accompanying gestures are categorized into various types, based on the type of sentences. The following section looks at one of the most well-known categorizations of gestures.

## 3. A Classification of Gestures Accompanying Speech

McNeill [14] classifies gestures into four main categories: pointing (deictic) gestures, iconic gestures, metaphoric gestures, and beat gestures. Pointing gestures are used to show the location of objects to which they refer. For example, when talking about a house, one may use an extended index finger to refer to that house. There is no semantic relationship between the shape of pointing gestures and the physical characteristics of the objects they refer to. They merely show the location of objects in the environment. Iconic gestures are used to depict the shapes of objects. This illustration may be shown by the shapes of hands, the trajectory of hand movements, or both. Iconic gestures have a meaningful semantic relationship with the objects they refer to. In other words, in an iconic gesture, the shape of hands and the trajectory of hand movements have a significant similarity with the physical characteristics of the objects they refer to. For example, the shape of hands and the trajectory of hand movements may be used to talk about the physical features of the wheel of a car. Metaphoric gestures are used to metaphorically describe a concept, an object, or an event. For example, the upward movement of a hand may be used to refer to the promotion of an individual in an organization. In this example, promotion in an organization is metaphorically described as an upward movement. In a metaphoric gesture, there is no literal or direct semantic relationship between the shape of hands (or trajectory of hand movements) and the physical characteristics of the concept, object, or event they refer to. Beat gestures occur in parallel with the prosody of speech. However, they do not have any meaningful semantic relationship with the accompanying speech and do not add any semantic content to the speech.

In this article, parallel with our classification of metaphoric and literal statements, we specifically focus on iconic and metaphoric gestures. The reason for choosing iconic and metaphoric gestures for our study was that these two types of gestures are related to the embodied meanings of the concepts they refer to. The other two types of gestures (iconic gestures and beat gestures) do not have a meaningful relationship with the embodied meanings of the concepts. Since we intended to study the embodied meanings of concepts, we did not include iconic gestures and beat gestures in our study. Before explaining the study’s methodology, we briefly review some works that have discussed iconic and metaphoric gestures as embodied simulations of literal and metaphorical descriptions.

## 4. Iconic and Metaphoric Gestures as Embodied Realizations of Mental Simulations

According to the Gesture as Simulated Action framework [15,16], gestures are embodied realizations of mental simulations. Based on this model, spatial and motoric properties are the key reason behind using gestures. In other words, people use gestures to present concrete or visually perceivable images of the spatial and motoric properties of objects, concepts, or events that are mentally simulated. Regardless of whether a particular thing or event is spatial/motoric or metaphorically described in terms of spatial/motoric properties, gestures may be used to describe it [16]. In the case of iconic gestures, the image of spatial/motoric properties of a concrete object is directly described in terms of body shapes (usually hand shapes) or the tracing of movements of body parts (usually hands). In the case of metaphoric gestures, a given concept (usually an abstract concept) is described in terms of a concrete object or a movement, and gestures are employed to depict that object or movement.

The Gesture as Simulated Action framework and embodiment theories share the view that gestures are embodied realizations of mental processes. Embodiment theories hold that the same neural networks involved in perceiving a concrete object or performing an action are activated when we talk or think about that object or action. It has been argued that this is even the case with metaphorical descriptions of concepts [11]. For example, when the metaphorical phrase *grasp an idea* is produced or comprehended by an individual, the same neural networks involved in performing the grasping action are activated. This means that the action of grasping is simulated when we talk or think about the action of grasping. Importantly, this could happen even in metaphorical talking or thinking. This mental simulation could be accompanied by a grasping gesture, or not accompanied by such a gesture.

Here, we report an experiment that aimed to investigate the gestural realizations of literal and metaphorical descriptions in the process of retelling a set of short stories by a group of participants. This experiment was based on our classification of metaphorical and literal statements. In this experiment, we compared the number of gestures used with each category of metaphorical statements and the number of gestures used with its corresponding literal category. The aim was to examine the possibility of any similarity or difference between gestural realizations of metaphorical and literal statements. This constituted a one-to-one comparison between metaphorical and literal corresponding categories in our proposed classification of metaphorical and literal statements. Following the embodiment view of cognition, we expected there to be similarities between the gestural or embodied realizations of corresponding categories.

## 5. Method

### 5.1. Stimuli

To elicit gestures, we employed a series of audio clips containing metaphorical and literal statements and asked the participants to retell the stories. The stimuli were suitable for our research since each story contained four metaphorical statements (one motion-based metaphor, one static space-based metaphor, one static object-based metaphor, and one static event-based metaphor) and four literal statements (one motion-based literal statement, one static space-based literal statement, one static object-based literal statement, and one static event-based literal statement). The researchers designed these clips for the purpose of this study. Each short story was an audio recording of about seven minutes in length and included 600–650 words. The stories were titled “Perseverance and Success”, “A Successful Businessman”, “Tomorrow Is Bright”, “Escape in the Darkness”, and “Meeting in the Rain”. The order of metaphorical and literal statements was randomized across the five stories (see the English version of the metaphors and literal statements in Appendix A).

### 5.2. Participants

The participants of this study were randomly selected from a large population of students at Chabahar Maritime University, according to convenience sampling as a subtype of non-probability sampling. The reason for this is that we did not want to control the population of the study for any particular feature. We preferred to have a heterogeneous group of participants from Chabahar Maritime University, in order to prevent our data skewing towards a specific sample of society and to ensure that our study is generalizable. The sample size consisted of 30 bachelor’s students. Since gender and age were not considered variables in this study, participants were selected regardless of their age and gender. All participants were Persian native speakers.

### 5.3. Design and Procedure

We deployed a quantitative experimental research design with a gesture elicitation event. To prepare the participants for retelling the stories, we held a training session to prepare the participants for the main experiment of the study. The researchers did not perform any storytelling to avoid the observer-expectancy effect; only the steps to be taken by the participants were instructed. In this session, the participants listened to two sample stories. After listening to each sample, they had to retell the stories in front of a camera. The study’s primary goal was not revealed (to prevent courtesy and sucker biases) as it could affect the participants’ performance in the study’s main experiment. They were told to retell each story in their own words and mention as many details as they could remember. Before conducting the main experiment, the participants were provided with clear oral instructions to ensure they were completely prepared. Each participant sat in front of a computer screen. The audio recording of the first story was played in around seven minutes. After listening to the story, the participants had to turn on the cameras on their screens and retell the story in their own words. The participants were given seven minutes to retell the story. The camera’s location allowed it to record the participants’ gestures while they were retelling the story. The researchers were not present at the recording session to eliminate the potential Hawthorne effect. The same procedure was used for the other four stories in the experiment. All participants listened to the stories in the same order.

### 5.4. Data Analysis

The data provided from the video recordings were transcribed, classified, and quantified to determine the number of metaphorical and literal statements used during storytelling. Subsequently, the number of metaphoric gestures used with each category of metaphors and the number of iconic gestures used with each category of literal statements were obtained. The gestures produced by participants while retelling the stories were closely examined to ensure that only metaphoric and iconic gestures were selected for further analysis, whereas pointing gestures and beat gestures were removed from the analysis. For metaphorical statements used during the retelling of the stories, chi-square data produced through a contingency table analysis were used to compare the number of metaphoric gestures used with the four categories of metaphorical statements. The aim was to examine the possibility of any similarity or difference between the number of gestures accompanying the four categories of metaphorical statements. A similar analysis was conducted for literal statements. An independent t-test was administered to directly compare each category of metaphorical statements and its corresponding literal category. In this analysis, the percentages of cases where gestures accompanied statements of each category were compared with one another.

## 6. Results

The numbers of produced metaphorical and literal statements are given in Table 2 and Table 3. These numbers are given for each type of metaphorical and literal statement separately. The number of produced metaphoric and iconic gestures accompanying each category of metaphorical and literal statements during retelling stories is given in the third row of these tables.

The results of a contingency table analysis showed that, among the four categories of metaphorical statements, the participants used the smallest number of metaphoric gestures with static event-based metaphors (χ^2^ = 28.77, *p* < 0.00001). The results of another contingency table analysis showed that, among the four categories of literal statements, the participants used the smallest number of iconic gestures with static event-based literal statements (χ^2^ = 23.00, *p* < 0.0004). These results indicate a similarity between the patterns of using gestures (metaphorical and iconic) and statements (metaphoric and literal). In both groups of metaphorical and literal statements, static event-based statements were accompanied with the smallest number of gestures.

In addition to this analysis, the percentage of gestures used with each category of statements was calculated. These results are presented in Table 4.

These results show that there is a noticeable similarity between each category of metaphorical statements and its corresponding literal category in terms of the percentage of gesture usage with each category of statements. A T-test was used to make a comparison between the percentages related to metaphorical and literal statements. The *p*-value was 0.96. The means of percentages for metaphorical and literal statements were 63.5 and 62.5, respectively. These show that percentages related to metaphorical and literal statements were significantly similar. This similarity was more noticeable with motion-based, static object-based, and static-event-based statements.

## 7. Discussion

As mentioned in the previous section, among the categories of metaphorical and literal statements, static event-based metaphors and static event-based literal statements were accompanied by the smallest number of gestures. One possible explanation for this is that the meanings of such statements are less strongly associated with space and motion, compared to the other categories of metaphorical and literal statements [15,16]. Gestures are often used to provide information about spatial and motoric properties. Motion-based metaphors and motion-based literal statements refer to a motion event. Since motion is the key part of the meaning of such statements, gestures can be effective tools to provide more information about the meanings of such statements. Regardless of whether the information about a motion is fully or partially expressed in speech, gestures can be effectively used to provide visual and motoric information. Therefore, gestures may provide redundant or necessary (complementary) information. In the case of providing redundant information, gestures express information that is expressed in the accompanying speech. In the case of providing necessary (complementary) information, gestures express information that is not expressed in the accompanying speech.

The meanings of motion-based statements (metaphorical or literal) have strong visual and motoric components. Other modalities (hearing, olfactory, haptic, gustatory) often do not have a prominent role in motions. This is also the case with gestures, as gestures are usually less strongly associated with non-visuo-motor modalities. Gestures are effective tools for expressing visual and motoric information. Therefore, they are practical tools to be aligned with motion-based metaphors and motion-based literal statements.

A similar argument can be made about the high number of gestures that were used with static space-based metaphors and static space-based literal statements. Since spatial positions of objects are the central part of the meanings of such statements, gestures are used with them to highlight this key part. For example, in the metaphor *she was always at the top among her classmates* and in the literal statement *the rope was over the wall*, the central meaning concerns the location of something in space. This central part can be clearly highlighted by a gesture. Static object-based statements differ from the previous two categories in that such statements are primarily based on a concrete object. The spatial and motoric properties of this object may or may not be an essential part of the meaning of such statements. Therefore, depending on the extent to which the spatial and motoric features of the object are important, gestures may or may not be used with these statements. If the spatial and motoric features of the object are important to the meaning of the statement, there is a high possibility that a gesture will accompany the statement. If the spatial and motoric features of the object are not necessary to a statement’s meaning, that statement is less likely to be accompanied by a metaphoric or iconic gesture. In effect, the use of gestures is contingent on importance. If a certain aspect of meaning is crucial, its expression may be supported by a gesture. This is especially true when that crucial part is spatial and motoric. Static event-based statements (metaphorical or literal) are noticeably different from the other categories, in that spatial and motoric properties have the lowest level of importance in such statements. As mentioned above, spatial and motoric elements may be involved, to some degree, in the meanings of such statements, but these elements are peripheral to the primary intended meaning. This could explain why static event-based statements (metaphorical or literal) have a relatively low tendency to be accompanied by gestures.

**Figure 1:**
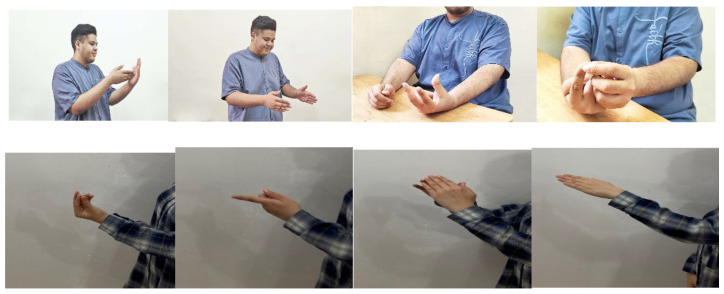
Some examples of the gestures that accompanied literal and metaphorical statements in the study. Note: the photos were taken by the researchers, and publishing permission was obtained from the participants.

Another noticeable finding of this study was the significant similarity of the percentages of gesture usage between each category of metaphorical statements and its corresponding literal category. These results support the assumptions of the strong version of embodiment [11]: for a review, see [17]. These results suggest that each category of metaphors and its corresponding literal category are embodied in the same way. As mentioned above, it has been argued that the metaphorical phrase *grasping an idea* and its corresponding literal phrase *grasping an object* are embodied in the same way. That is, the same neural networks that are involved in the real action of grasping an object are actively recruited to understand or produce these phrases. The results reported in Table 3 are significantly in line with this view. This suggests that, in terms of mechanisms of embodied understanding, metaphorical and literal statements are similar [18,19,20,21,22]. Some examples of similarity between the embodied/gestural realization of each metaphorical category and the embodied/gestural realization of its corresponding literal category have been shown in Figure 1.

According to the strong versions of [11], this similarity exists in the neural patterns that underlie concepts in a literal category and its corresponding metaphorical category. Importantly, this similarity may exist even in the absence of gestures.

The results given in Table 4 show that, among the four comparisons, the gap between static space-based metaphors and literal static space-based statements was more significant than the gap between the other corresponding categories. One possible explanation is that the individual may want to provide more detailed information about a concept/event in some cases. This information can be provided by gestures. The participants used the highest number of gestures with static space-based metaphors because they may have wanted to provide more detailed information about the concepts in their metaphorical statements.

## 8. Limitations of the Study and Future Directions

Because of the difficulty of accessing people from various cultures, the participants of the study were selected from among Iranian students. Culture is one of the key factors that may strongly influence gesture use. For example, this study did not investigate cross-cultural differences in gestures with various categories of metaphorical and literal statements [23]. Therefore, the scope to interpret the results is limited. Conducting cross-cultural studies on how gestures are used with various categories of metaphors and literal statements is one of the possible areas that can be investigated in future research projects [24]. For example, the possible similarities or differences between the ways that gestures are used with each category of metaphorical statements and its corresponding literal category is a question that can be examined in future works.

## 9. Conclusions

The results of this study support the view of embodied metaphor comprehension. The most novel finding of this study concerns the significant similarity between the ways each category of metaphor and its corresponding literal category is embodied in the form of gestures. However, the way that metaphorical and literal statements are embodied as gestural representations mainly depends on spatial and motoric associations. When the meaning of a metaphorical or literal statement has strong spatial and motor associations, the possibility of using gestures with that statement is higher. Interestingly, there is no significant difference between metaphorical and literal statements in this respect. This again supports the idea that the mechanisms underlying the embodiment of metaphorical and literal statements are essentially similar.

## Figures and Tables

**Table 1:** Two parallel classifications of metaphors and literal statements.

Classification of Metaphors	Classification of Literal Statements
Motion-based metaphors	Motion-based literal statements
Static space-based metaphors	Static space-based literal statements
Static object-based metaphors	Static object-based literal statements
Static event-based metaphors	Static event-based literal statements

**Table 2:** Total counts of produced metaphorical statements and metaphoric gestures for each category.

Category	Motion-Based Metaphors	Static Space-Based Metaphors	Static Object-Based Metaphors	Static Event-Based Metaphors
Total number of produced metaphorical statements	123	116	99	102
Total number of produced metaphoric gestures accompanying each category	98	96	71	23

**Table 3:** Total counts of produced literal statements and iconic gestures for each category.

Category	Motion-Based Literal Statements	Static Space-Based Literal Statements	Static Object-Based Literal Statements	Static Event-Based Literal Statements
Total number of produced literal statements	118	121	93	89
Total number of produced iconic gestures accompanying each category	96	88	69	21

**Table 4:** The percentages of gesture usage for each category.

**Category**	**Motion-Based**	**Static Space-Based**	**Static Object-Based**	**Static Event-Based**
Metaphorical	79%	82%	71%	22%
Literal	81%	72%	74%	23%

## Data Availability

Data sharing not applicable.

## Appendix A

English translations of the metaphors and literal statements used in the stories.

**Motion-based metaphors**

1. He rose up in the office of company’s manager
2. He passed through a difficult time to achieve his goals
3. The two former friends separated their ways of life and went into different directions
4. The company is going in the wrong direction
5. She had to pass through a storm to arrive at the land of her dreams

**Static space-based metaphors**

1. A large part of the population was struggling at the lowest levels of economic power
2. She was always at the top among her classmates
3. People who are at the center of power are unaware of the pains of the poor
4. His performance was above the expected level
5. The quality of her work was much higher than that of her rivals

**Static object-based metaphors**

1. She was faced with a mountain of obstacles
2. Some people were rigidly adhered to their old beliefs
3. The advice was a candle
4. Corruption was a tumor for the society
5. The college was the door to a new world for her

**Static event-based metaphors**

1. His dreams were destroyed by the disaster
2. That event was an earthquake in the company
3. The news was an explosion
4. The new inventions were a revolution
5. They cut their relationship

**Motion-based literal statements**

1. He climbed up the ladder
2. They climbed up the wall
3. She entered the room
4. The car was moving fast along the road
5. They were walking slowly

**Static space-based literal statements**

1. Her books were on the table
2. It was located on a hill
3. The building was beside a big garden
4. The rope was over the wall
5. The guard was near the gate

**Static object-based statements**

1. The building was large
2. The doors were irony and heavy
3. The street was full of customers
4. The walls were really high and strong
5. The road was dark and wet

**Static event-based statements**

1. The candle was burning
2. A large tree fell on the road
3. A fire in the factory caused a lot of damages
4. The sirens warned the guards
5. The wind caused a famine in the area
